# RIG-I recognizes metabolite-capped RNAs as signaling ligands

**DOI:** 10.1093/nar/gkad518

**Published:** 2023-06-16

**Authors:** Brandon D Schweibenz, Mihai Solotchi, Pranita Hanpude, Swapnil C Devarkar, Smita S Patel

**Affiliations:** Department of Biochemistry and Molecular Biology, Robert Wood Johnson Medical School, Rutgers University, Piscataway, NJ 08854, USA; Graduate School of Biomedical Sciences at the Robert Wood Johnson Medical School of Rutgers University, USA; Department of Biochemistry and Molecular Biology, Robert Wood Johnson Medical School, Rutgers University, Piscataway, NJ 08854, USA; Graduate School of Biomedical Sciences at the Robert Wood Johnson Medical School of Rutgers University, USA; Department of Biochemistry and Molecular Biology, Robert Wood Johnson Medical School, Rutgers University, Piscataway, NJ 08854, USA; Department of Biochemistry and Molecular Biology, Robert Wood Johnson Medical School, Rutgers University, Piscataway, NJ 08854, USA; Graduate School of Biomedical Sciences at the Robert Wood Johnson Medical School of Rutgers University, USA; Department of Biochemistry and Molecular Biology, Robert Wood Johnson Medical School, Rutgers University, Piscataway, NJ 08854, USA

## Abstract

The innate immune receptor RIG-I recognizes 5′-triphosphate double-stranded RNAs (5′ PPP dsRNA) as pathogenic RNAs. Such RNA-ends are present in viral genomes and replication intermediates, and they activate the RIG-I signaling pathway to produce a potent interferon response essential for viral clearance. Endogenous mRNAs cap the 5′ PPP-end with m^7^G and methylate the 2′-O-ribose to evade RIG-I, preventing aberrant immune responses deleterious to the cell. Recent studies have identified RNAs in cells capped with metabolites such as NAD^+^, FAD and dephosphoCoA. Whether RIG-I recognizes these metabolite-capped RNAs has not been investigated. Here, we describe a strategy to make metabolite-capped RNAs free from 5′ PPP dsRNA contamination, using *in vitro* transcription initiated with metabolites. Mechanistic studies show that metabolite-capped RNAs have a high affinity for RIG-I, stimulating the ATPase activity at comparable levels to 5′ PPP dsRNA. Cellular signaling assays show that the metabolite-capped RNAs potently stimulate the innate antiviral immune response. This demonstrates that RIG-I can tolerate diphosphate-linked, capped RNAs with bulky groups at the 5′ RNA end. This novel class of RNAs that stimulate RIG-I signaling may have cellular roles in activating the interferon response and may be exploited with proper functionalities for RIG-I-related RNA therapeutics.

## Graphical Abstract

**Graphical Abstract:**
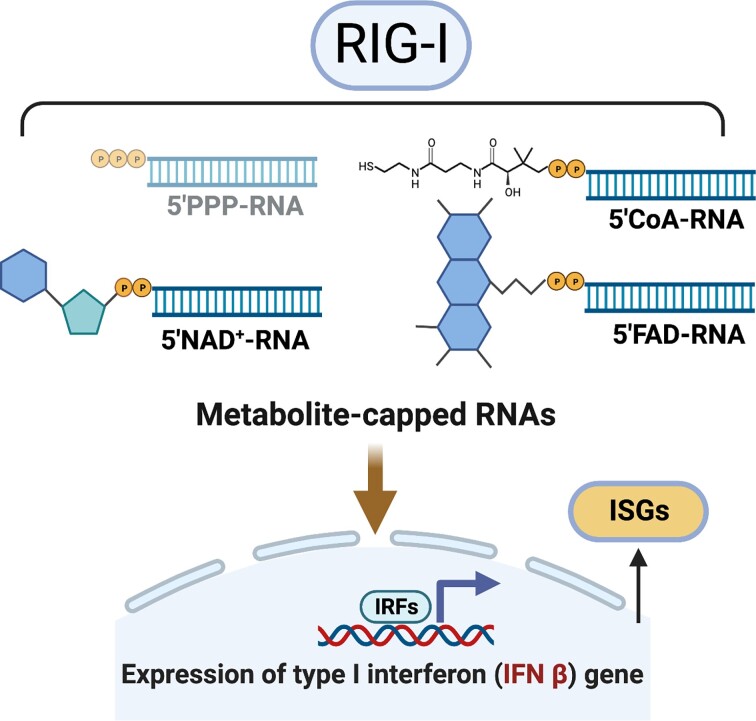

## INTRODUCTION

RIG-I (Retinoic Acid Inducible Gene-I) is a pattern recognition innate immune receptor that belongs to the DEA(X)D-box family of helicases. Its primary function is to surveil the cytoplasm for non-self RNAs arising from infecting viruses and other pathogens (1). RIG-I recognizes blunt-ended double-stranded (ds) RNAs that contain either 5′-triphosphate (5′ PPP) or 5′-diphosphate (5′ PP) ends as pathogen-associated molecular patterns (PAMPs) (2, 3). These features are hallmarks of viral genomes and replication intermediates of negative-strand and positive-strand RNA viruses (4, 5). Upon recognizing PAMPs, RIG-I initiates a signaling cascade culminating in a Type-I interferon response that controls the viral infection and primes the adaptive response (5–8).

**Figure 1.**
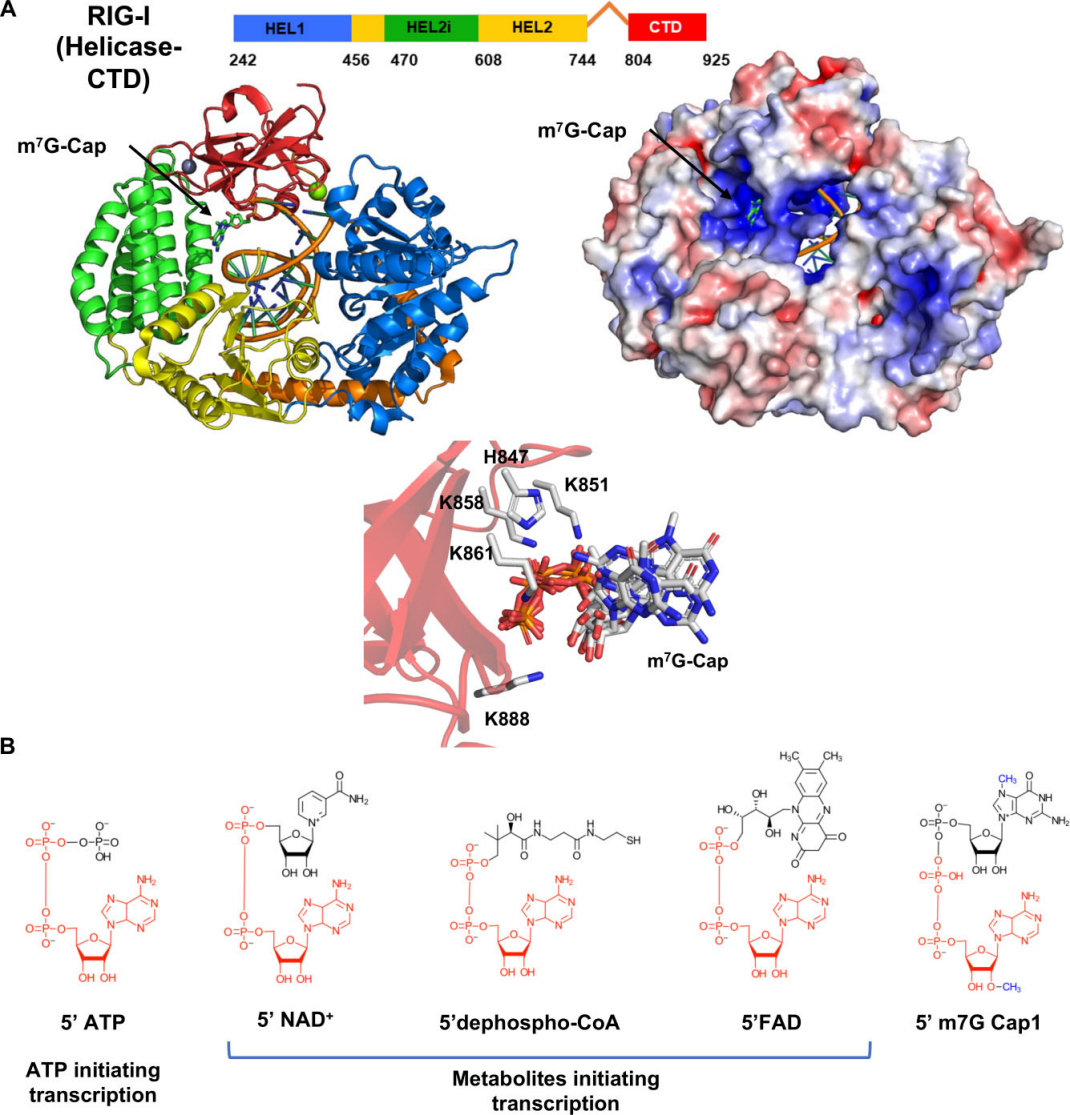
Structures of the RIG-I complex with m7G Cap0 RNA and ADP-based metabolites in metabolite-capped RNAs. (**A**) The domain structure of RIG-I Helicase-CTD and the crystal structure of the Cap0 RNA-bound RIG-I Helicase-CTD (PDB ID: 5F98) are shown in the same orientation as the cartoon and space-filled models. The RIG-I domains are color-coded. The middle panel shows the zoomed view of CTD residues interacting with the triphosphate in Cap0. Oxygen atoms are colored red, phosphorus orange, nitrogen blue, and carbon white, while the CTD is transparent red. Six members of the asymmetric unit are aligned and overlaid. The triphosphate linkage demonstrates little differences between asymmetric units, while the Cap0 moiety, which is much bulkier, twists in multiple different conformations without contacting RIG-I residues. (**B**) The structure of ATP and the metabolites incorporated at the 5′-end of RNA as 5′-PPP adenosine, 5′ NAD^+^, 5′ dephospho-CoA, and 5′ FAD are shown. The red color highlights the ADP-based moieties serving as the initiating nucleotide for transcription. All metabolite cap moieties used here have been identified in bacterial RNA transcripts, while only NAD+ or NADH-capped RNAs have been identified in humans and yeast. The eukaryotic m7G Cap1 structure has the methyl groups of G-cap and 2′-position of the first ribose highlighted in blue. Combining the G-cap and the 2′ methylation is essential for RIG-I evasion.

In eukaryotes, the 5′ PPP ends of the mRNAs are capped with 7-methyl guanosine (m^7^G), which can affect RIG-I’s ability to bind cellular RNAs. Previous studies have shown that RIG-I binds m^7^G Cap0 RNA comparably to 5′ PPP RNA; however, the combination of the m^7^G cap and a methylation event at the 2′ position of the first ribose, together called Cap1, reduces RIG-I affinity by approximately 200-fold (9, 10). Structural studies show that RIG-I’s C-terminal domain (CTD) contacts the triphosphate linkage of Cap0 but does not interact with the m^7^G cap moiety, and there is space in the RNA-bound RIG-I complex to accommodate the cap (Figure 1A) (9). Both Cap0 and Cap1 RNAs are translated in the cell, but many viruses, particularly flaviviruses, have evolved strategies to chemically synthesize mRNA with Cap1 moiety for immune evasion (11–14).

RNA caps are not limited to eukaryotes; RNAs capped with metabolites such as NAD^+^ and Coenzyme A were initially discovered in bacteria (15, 16). Since then, additional alternative caps like FAD, UDP-Glc and UDP-GlcNac have been identified in various organisms, such as yeast, plants, and human mitochondria, and cultured Dengue virus genomes (17–20). These alternative caps are of different sizes: the NAD^+^ is similar to the m^7^G canonical cap, dephosphoCoA has a long hydrocarbon chain, and FAD has a bulky, three-membered ring structure (Figure 1B). Capping with metabolites occurs during transcription initiation when metabolites are incorporated instead of initiating NTP. For example, the ADP-based metabolites can compete with ATP for transcription initiation, get incorporated at the 5′-end of RNA, and are thus referred to as noncanonical initiating nucleotides (NCINs) (21). While no capping enzymes for metabolites have been identified, initiation may not be the only mechanism for alternative capping. Small nucleolar RNAs (snoRNAs), introns generated through RNA splicing, have been identified with NAD^+^-caps, suggesting that some post-transcriptional capping mechanism may exist (20). The role of these caps is not yet well defined. The relative amount of NAD^+^-cap versus NADH-cap in the mitochondria is sensitive to the metabolite concentrations (19). In cytoplasmic contexts, the NAD^+^-capped RNAs are not translated, but NAD^+^ capping is thought to promote RNA turnover (20). Because RIG-I recognizes Cap0 RNAs, we were curious to know if these metabolite-capped RNAs found in the cell are immunogenic through the RIG-I activation pathway.

An important difference between the metabolite-capped RNAs and a comparable Cap0 RNA is the phosphate linkage between the cap and the first nucleotide. In each alternative cap, the metabolite moiety is connected to adenosine with a diphosphate linkage. In contrast, in Cap0 and Cap1, the guanosine cap is connected to the first nucleotide through a triphosphate linkage (Figure 1B). RIG-I predominantly interacts with the α- and β-phosphates of Cap0 RNA while making a single contact with the γ-phosphate of Cap0 RNA (3, 9). The 5′ PP RNAs are also recognized as PAMPs (2), suggesting that γ-phosphate recognition is dispensable. However, the difference in length between the di- and tri-phosphate linkages may be relevant to how the cap moiety positions itself in the CTD binding pocket. The short di-phosphate link would bring the cap moiety closer to the CTD and potentially hinder phosphate interactions.

Our initial guess was the shortened phosphate linkage, combined with the bulky cap, would inhibit RIG-I recognition. However, this study shows that RIG-I recognizes the metabolite-capped dsRNAs derived from various metabolites similar to 5′ PPP and Cap0 RNAs. Using *in vitro* transcribed metabolite-capped RNAs, we demonstrate that RIG-I binds to metabolite-capped RNAs in an ATPase-competent manner and utilizes these RNAs as oligomerization scaffolds and ultimately as signaling ligands in cell signaling reporter and antiviral signaling assays. That RIG-I recognizes metabolite-capped RNAs of significantly different geometries demonstrates its tolerance for a wide variety of 5′ phosphate-linked moieties and constrains how such RNAs can appear in the cytoplasm without generating an immune response.

## MATERIALS AND METHODS

### RNA transcription and purification

5′PPP and 5′metabolite-capped 39-mer ssRNAs were transcribed *in vitro* using a bacteriophage T7 RNA polymerase system. 400 μl transcription reactions were run for 3 h at 30**°**C after mixing all the necessary reaction components, including 250 mM HEPES pH 7.5 buffer, 30 mM MgCl_2_, 40 mM DTT, 2 mM spermidine, 5 μg inorganic pyrophosphatase, 50 U RNasin, 2 μM dsDNA promoter and template sequence (Supplemental Table S1), 5 mM GTP and UTP, 8 mM ATP or desired metabolite (NAD^+^, Roche; dephospho-CoA, Sigma; FAD, Sigma), and 1 μM T7 RNA polymerase, purified as previously described (22). Reactions were quenched with 75 mM EDTA.

The 39-mer ssRNA products were purified by gel electrophoresis on 1.5 mm × 30 cm 40% acrylamide and 6 M urea gel in 1.5× TAE. Electrophoresis was conducted at 500 V for 4 h, and the resulting ssRNA was identified by UV shadowing, the band was excised, and RNA was extracted from the gel using a Whatman Elutrap electroelution system in 1.5× TAE buffer run at 150 V for 6 h. The resulting ssRNA was concentrated with ethanol precipitation and stored at –80°C.

*In vitro*, synthesized ssRNAs were analyzed for purity and metabolite cap incorporation using LC–MS (Novatia, LLC; Newton, PA). Samples were run at a 200**°**C source temperature rather than 350–375**°**C to prevent RNA 5' caps detachment.

The fluorescently labeled ss39 complementary ssRNA and the 5′ OH 5′ OVG ssRNA were chemically synthesized (Dharmacon).

### Protein expression and purification

The RIG-I gene was cloned in the protein expression vector pET28 SUMO and expressed as SUMO fusion proteins in *Escherichia coli* strain Rosetta (DE3) (Novagen). The protein was purified using a series of chromatography columns as published previously (3). The soluble lysate was fractionated through a HisTrap HP (GE Healthcare), followed by Ulp1 protease digestion to remove 6× His-SUMO tag, hydroxyapatite (CHT-II, Bio-Rad), and heparin Sepharose (GE Healthcare). The purified protein was dialyzed into 50 mM HEPES pH 7.5, 50 mM NaCl, 5 mM MgCl_2_, 5 mM DTT and 10% glycerol overnight at 4°C, frozen in liquid nitrogen and stored at –80°C.

### RNA *K*_D, app_ measurements using ATP hydrolysis-based titrations

ATP hydrolysis was measured at constant RIG-I (15 nM) and increasing RNA concentration (1–400 nM) in the presence of 2 mM ATP (spiked with [γ-^32^P] ATP). The ATPase reaction was measured after 20, 40, and 60 min of reaction times in ATPase buffer (50 mM MOPS pH 7.4, 5 mM DTT, 5 mM MgCl_2_, 0.01% Tween20) at 25°C. Reactions were stopped at each time point using 4 N formic acid and analyzed by PEI-Cellulose-F TLC (Merck) developed in 0.4 M potassium phosphate buffer (pH 3.4). TLC plates were exposed to a phosphorimager plate, imaged on a Typhoon phosphor-imager, and quantified using the ImageQuant software. The molar [Pi] generated during the reaction time intervals was plotted against time and fit to a linear equation with 3 points, not including 0 min. The slopes (ATPase rate) were plotted as a function of RNA concentration and fitted to the following equation to obtain the RNA *K*_D,app_ values: slope = *k*_atpase_ × [PR]/[Pt]; where [Pt] is total protein concentration; [PR] is the amount of RIG-I/RNA complex formed and [R] is the RNA concentration being titrated. Each point's associated error was calculated from the linear fit. The quadratic equation (Equation 1) was used to determine [PR] and estimate the *K*_D,app_ and *k*_atpase_. Reported errors for *K*_D,app_ and *k*_atpase_ are fitting errors.


(1) \begin{eqnarray*} \left[ {PR} \right] = \frac{{\left( {\left[ {{P}_t} \right] + \left[ {{R}_t} \right] + {K}_{D,\ app}} \right) - \sqrt {\left( {\left[ {{P}_t} \right] + \left[ {{R}_t} \right] + {K}_{D,\ app}{)}^2 - 4[{P}_t} \right]\left[ {{R}_t} \right]} }}{2}\ \end{eqnarray*}

### Fluorescence polarization assays

Fluorescence polarization measurements were performed on a Tecan Spark microplate reader in a 384-well black plate at 25°C. A monochromator set the excitation wavelength at 485 nM and the emission wavelength at 535 nm, with a 20 nm bandwidth. Purified RIG-I protein was serially titrated in 1× ATPase buffer (50 mM MOPS pH 7.4, 5 mM DTT, 5 mM MgCl_2_, 0.01% Tween20) and incubated with a constant 20 nM fluorescein-labeled dsRNA for 15 min at 25°C; 500 μM ATP was added prior to taking measurements.

To obtain *K*_D_ values for each RIG-I’s binding affinity to each RNA, polarization values from triplicate data sets were plotted as a function of protein concentration. The data were fit to equations 2 and 3. The observed fluorescence polarization (*F*) from the initial fluorescence polarization (*F*_0_) is proportional to the amount of protein–RNA complex (*PR*) and modified by a coefficient of complex formation (*f*_c_). Reported errors for *K*_D_ are fitting errors.


(2) \begin{eqnarray*}F\ = {F}_0\ + {f}_c * [PR]\end{eqnarray*}

(3) \begin{eqnarray*}{\mathrm{\ }}\left[ {PR} \right] = \frac{{\left[ P \right]}}{{{K}_D + \left[ P \right]}}\ \end{eqnarray*}

To measure the affinity of ssRNA, we used a competition binding assay where the unlabeled ssRNA was serially titrated in 1× ATPase buffer and incubated with a constant, pre-mixed 50 nM RIG-I and 20 nM fluorescent dsRNA for 15 min at 25°C. 500 μM ATP was added prior to taking measurements under the same conditions previously stated. Polarization values were plotted as a function of ssRNA concentration. Unlabeled dsRNA was used as a control to demonstrate competition for RIG-I binding against the fluorescent dsRNA. This data was fit to equations (2) and (3) to estimate the IC_50_ value rather than *K*_D_.

### Electrophoretic mobility assays (EMSA)

EMSAs were performed by incubating RIG-I (75 nM) and ds39 RNAs (25 nM) in ATPase buffer for 60 min at 4°C. 2 mM ATP was added to each RIG-I containing reaction 15 min before loading the sample into the gel. Loading buffer (10× concentration of 1.5% Ficoll 400 in Tris-borate buffer, pH 8.0) was added to the samples loaded on a 4–16% gradient Native PAGE gel (Invitrogen) at 4°C. Gels were scanned at 532 nm using a Typhoon FLA 9500 laser-based scanner (GE).

### IFN-β reporter cell reporter signaling assays

HEK293T cells were grown in 5% CO_2_ and 37°C, in DMEM with 10% FBS in 6-well plates to 60% confluence and cotransfected with firefly luciferase reporter plasmid (pLuc125/2.5 μg), Renilla luciferase reporter plasmid (pRL-TK / 500 ng), and a plasmid carrying myc-tagged WT RIG-I gene under the constitutively active CMV promoter (pcDNA-3.1/2 μg). The firefly luciferase gene is under the control of the interferon β promoter, and the Renilla luciferase plasmid is under the control of the constitutively active TK promoter. The plasmid transfections were carried out with X-tremeGENE HP DNA Transfection Reagent (Roche). Cells were replated in 96-well plates the next day at 2 × 10^4^ cells/well density and transfected with indicated single-stranded and double-stranded RNAs (700 nM, or as indicated, in 110 μl volume) using Lyovec transfection reagent (InvivoGen). After 20 h, the activities of firefly and Renilla luciferases were measured sequentially with the Dual-Luciferase reporter assay (Promega). Each trial's number of replicates are shown in figure legends. Error bars represent the standard error of the mean (SEM).

### Isolation of RNA and quantitative real-time RT-PCR analysis

HEK293T RIG-I KO cells were grown in 5% CO_2_ at 37°C in DMEM cell culture medium (Gibco) with 10% FBS in 6-well plates to 80% confluence and transfected with empty vector and myc-tagged WT RIG-I gene under the constitutively active CMV promoter (pcDNA-3.1/2 μg). Cells were replated in 12 well plates the next day at 1 × 10^5^ cells/well density and transfected with indicated RNAs (50 nM in 110 μl volume) using 50 μl Lyovec transfection reagent (InvivoGen).

A549 and A549 RIG-I KO cells were maintained in RPMI cell culture Medium (Gibco) with 10% FBS. Cells were plated in 12-well plates at 1 × 10^5^ cells/well density and transfected with indicated RNAs (100 nM in 110 μl volume) using 50 μl Lyovec transfection reagent (InvivoGen).

Cells were washed with PBS, and total RNA was extracted from each well immediately using RNeasy micro kit (Qiagen, Hilden, Germany) 20 h post RNA transfection for HEK293T RIG-I KO and 25 h post RNA transfection for A549 and A549 RIG-I KO respectively. Cells were lysed by adding 350 μl of cold RLT lysis buffer containing 1% (v/v) 2-mercaptoethanol (MilliporeSigma) and passing the lysate through 1 ml Syringe 5–6 times. Total RNA was extracted from the lysate supernatant following the manufacturer's specification. The purification included in-column DNase treatment using the RNase-free DNase Set. The yield and purity of the RNA were measured using a NanoDrop 2000c spectrophotometer (ThermoFisher Scientific, Waltham, MA). The total RNA extracted ranged between 708 ng/μl to 1540 ng/μl concentration (Supplemental Figure S6B).

Complementary DNA (cDNA) was prepared using 1.5 μg of RNA using the High-Capacity cDNA Reverse Transcription Kit (Applied Biosystems, Carlsbad, CA) in a total volume of 20 μl reaction mixture following the manufacturer's specification.

The RT-qPCR analysis was performed following the MIQE guidelines http://rdml.org/miqe.html (23). Quantitative real-time PCR of specific genes was performed using the Platinum SYBR Green qPCR SuperMix-UDG with ROX (ThermoFisher Scientific, Waltham, MA) and primers specific for human IFNB1, ISG15, OAS1 or GAPDH (Supplemental Table S1) on a QuantStudio™ 3 Real‐Time PCR System (96‐well, 0.2 ml Block, Applied Biosystems, Waltham, MA). The incorporation of SYBR Green dye into the PCR products was monitored in real-time after each PCR cycle, resulting in the calculation of the threshold cycle or Ct value that defines the PCR cycle number at which exponential growth of PCR products begins. PCR cycle conditions were as follows: 2 min at 50°C, 10 min at 95°C, 40 cycles of 15 seconds at 95°C and 1 min at 60°C. To ensure no contamination, each PCR procedure included a negative control reaction without a template. Real-time PCR data were analyzed using the QuantStudioTM Design and Analysis Software V1.5.1 (Applied Biosystems, Waltham, MA). The GAPDH housekeeping gene was used as a reference for the relative quantification of the gene of interest, which was expressed as the ratio of the ‘concentration of the target’ to the ‘concentration of GAPDH’. Fold changes for RT-qPCR were determined by the ΔΔCT method. Data represent the mean of triplicate of one representative analysis.

Two biologically independent experiments were performed. Data represent the mean of triplicate of one representative analysis.

### Immunoblot analysis

Total protein lysates were prepared by lysing cell pellets in 2× SDS sample buffer (62.5 mM Tris, pH 6.8, 1% SDS, 15% glycerol, 2% β-mercaptoethanol and 0.005% bromophenol blue), and then boiled for 10 min. Proteins were separated by SDS PAGE and transferred onto 0.2 μm PVDF membrane. The membrane was immunoblotted with anti-RIG-I (1:500, CST D14G6 Rabbit mAb #3743S) and anti-actin (1:2500, CST 8H10D10 Mouse mAb #3700S) antibodies at 4°C overnight, followed by HRP-conjugated secondary antibodies (1:10 000, goat anti-rabbit or goat anti-mouse IgG) for 1 h. Blots were developed by using ECL western blotting substrate (ThermoFisher, Pierce) on a Biorad ChemiDoc system.

## RESULTS

### Generation of pure metabolite-capped RNAs through *in vitro* transcription using T7 RNA polymerase

RIG-I is potently stimulated by 5′ PPP RNAs. Therefore, it is critical to ensure that metabolite-capped RNAs synthesized enzymatically from *in vitro* transcription reactions are not contaminated with 5′ PPP RNA-ends. Previously published techniques of T7 RNA polymerase (T7 RNAP)-based transcription relied on purifying ADP-based metabolite-capped RNAs from 5′ PPP RNA (24). Because the two RNAs migrate close on gel electrophoresis, the purified metabolite-capped RNAs may still contain 5′ PPP RNA, which would be recognized by RIG-I and lead to false positive results (25).

**Figure 2.**
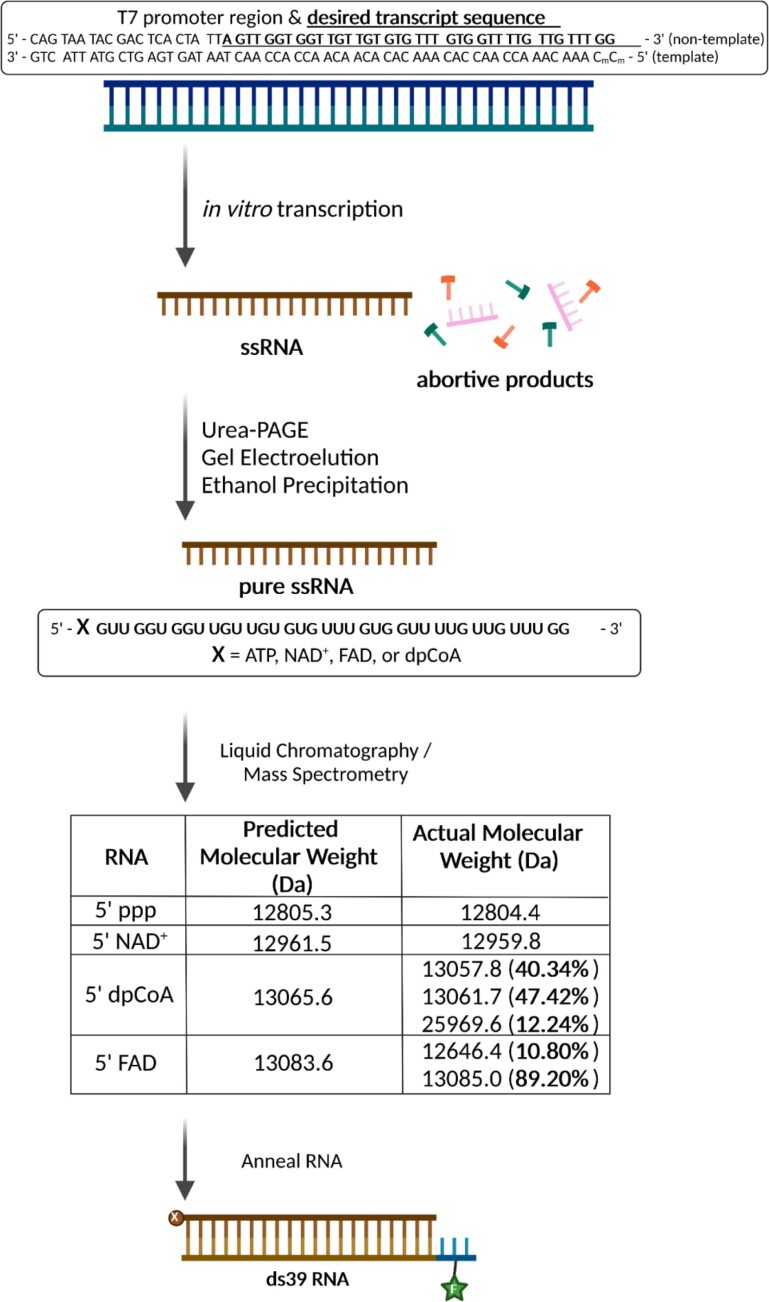
Synthesis of metabolite-capped RNAs by *in vitro* T7 transcription. T7 RNA polymerase promoter sequence and the desired transcript sequence (underlined) with adenine at the + 1 transcribing position. The sequence lacks internal adenosines, allowing transcription in the absence of ATP. The terminal methylated cytosines encourage T7 RNA polymerase dissociation from the promoter DNA upon completion of one round of RNA synthesis. Cytosines are excluded from the transcript sequence, and CTP is not added to the reaction to prevent copy-back transcription. The dsDNA promoter is incubated with T7 RNA polymerase and other reagents described in the Methods to generate the desired 39-mer RNA transcript, purified from remaining NTPs and abortive products using Urea-PAGE, gel electroelution, and ethanol precipitation. The purity of these ssRNAs was assessed using LC–MS, shown in the table, with percentages of each RNA species in parenthesis. If no percentage is shown, the purity is 100%. The ssRNAs were annealed to a chemically synthesized, complementary RNA with a 3-nt DNA overhang at the 5′-end to prevent RIG-I binding at the opposite end from the 5′PP-cap end, as well as having a fluorescein moiety on the second DNA position for biochemical assays. Created with Biorender.com.

Here, we describe an *in vitro* transcription method that would generate ADP-based capped RNAs without contaminating 5′ PPP RNAs. We designed a T7 DNA promoter with an initiating adenosine and 39 bp coding sequence that lacked internal A’s (Figure 2, Supplemental Figure S1). The 39 bp RNA length was chosen because it robustly stimulates RIG-I signaling activity compared to shorter RNAs (Supplemental Figure S1D). The initiating adenosine allows ADP-based metabolite incorporation at the + 1 position to form an alternative cap (Figure 1B). Because there are no internal A’s in the coding sequence, we could exclude ATP from the transcription reaction cocktail, preventing 5′ PPP contamination. We also excluded internal C’s and ended the RNA transcript with a terminal GG to avoid copyback synthesis, where T7 RNAP utilizes the nascent RNA transcript as a template to generate a duplexed RNA structure (26, 27). Excluding CTP from the reaction cocktail ensures a precise 3′ end and no contaminating, self-annealing RNAs. Additionally, we methylated the terminal CC of the promoter DNA template strand, which promotes T7 RNAP dissociation, again minimizing the copyback transcription (28). The transcribed RNA products were gel-purified, electro-eluted, and concentrated using ethanol precipitation.

The RNA sample purity was assessed by mass spectroscopy (MS, Figure 2, Supplemental Figure S2). The results confirmed the absence of 5′ PPP or 5′ PP contamination in the transcribed 5′ metabolite RNAs. The LC–MS analysis shows that 5′ PPP and 5′ NAD^+^ dsRNAs were nearly 100% pure. The 5′ dephosphoCoA and 5′ FAD capped RNAs contained small amounts of other mass species but not 5′ PPP or 5′PP. The 5′ dephosphoCoA capped RNA sample contained 12% of a species twice the expected molecular weight, corresponding to an RNA dimer from disulfide-linkage between the terminal sulfur groups of two dephosphoCoA moieties. The 5′ FAD capped RNA contained a peak at 12646.4 Da comprising 11% of the total peak area, close in mass to a 5′ monophosphate RNA (12639.3 Da), resulting from loss of the flavin moiety and one phosphate from the diphosphate linkage. These species could have been generated during the mass spectrometry analysis steps. RIG-I does not recognize the 5′ monophosphate RNA end, and if present in the sample, it should not generate a false positive (29). Aside from a disulfide-bridge mediated dimer with dephosphoCoA-capped RNA, we did not observe species whose sizes would correspond to incorrect 3′ extension by T7 RNAP copyback, indicating our transcription method produced precise 3′ ends.

Transcribed RNAs were annealed to a chemically synthesized, complementary ssRNA to generate 39-bp 5′PPP and metabolite-capped dsRNAs. The complementary ssRNA contained a 5′ DNA overhang of 3-nt, distal to the capped or PPP 5′ end of interest, to prevent RIG-I from binding at the opposite RNA end (Figure 2, Supplemental Figure S1). For biochemical studies, a fluorescein moiety was added to the DNA overhang. An additional control RNA was chemically synthesized with a 2-nt overhang and a 5′ OH instead of a 5′ PPP or a cap. The 5′ OH, 5′ OVG RNA-end has a weaker affinity for RIG-I and shows a low signaling activity in a reporter assay (9), serving as a negative control for RIG-I binding.

### Innate immune receptor RIG-I recognizes metabolite-capped dsRNAs

**Figure 3.**
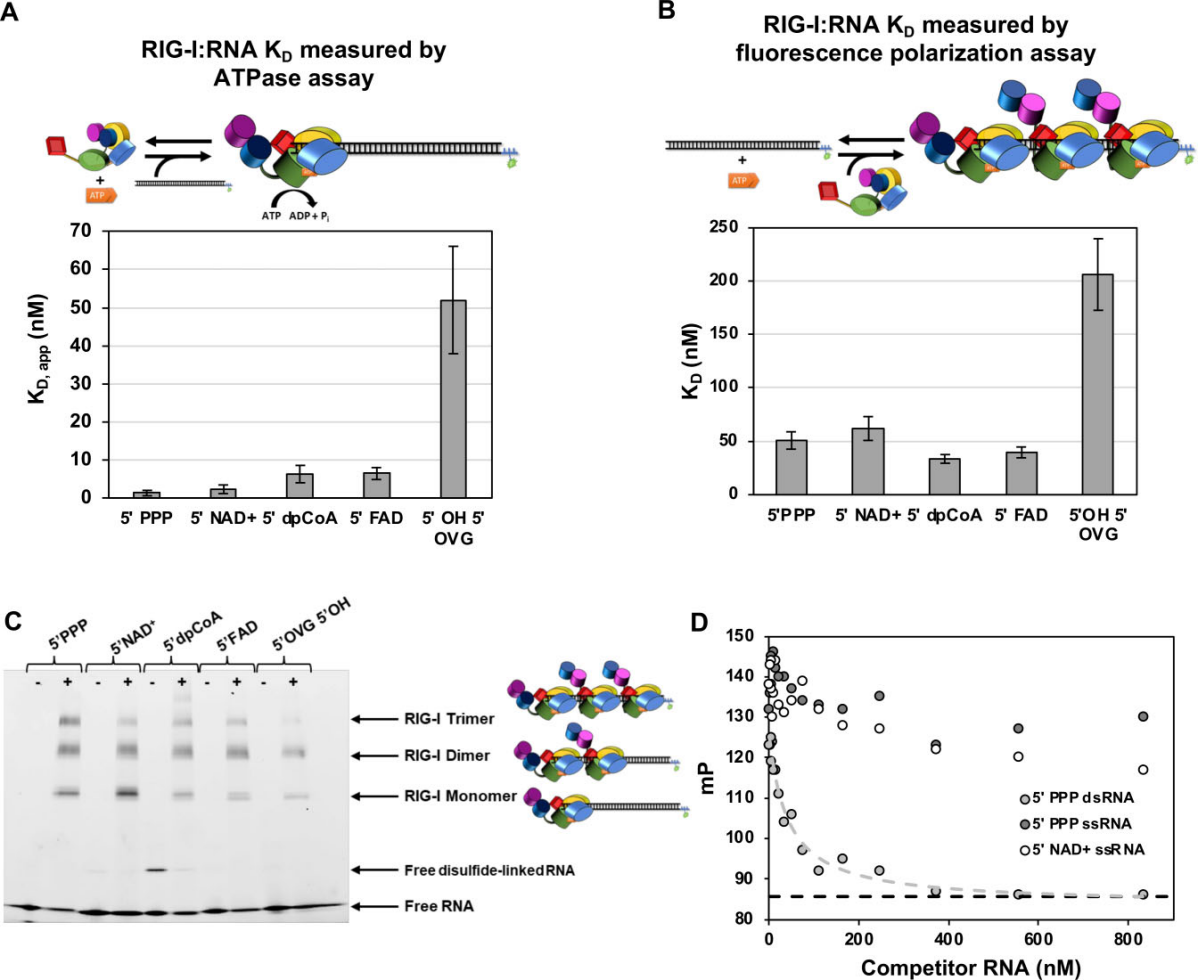
RIG-I binds to metabolite-capped RNAs comparably to 5′ PPP RNA. (**A**) Bar chart comparing *K*_D, app_ of RIG-I for various dsRNAs, measured using the RNA-dependent ATP hydrolysis assay. 15 nM of RIG-I was incubated with 2 mM ATP spiked with [γ-^32^P]-ATP, 1× ATPase Buffer, and increasing concentrations of either 5′ PPP ds39 RNA, 5′ NAD^+^ ds39 RNA, 5′ dephospho-CoA ds39 RNA, 5′ FAD ds39 RNA, or 5′ OH 5′ 2nt overhang RNA. The rate of ATP hydrolysis for each RNA concentration was measured as a time course at 0’, 20’, 40’, 60’ (*n* = 3) and fit with a linear equation. The average ATP hydrolysis rate was plotted as a function of RNA concentration and fit using a quadratic equation (Equation 1) to estimate the K_D,app_. The error bars are derived from the error of fit. Because of the high RNA concentrations relative to RIG-I, very likely RIG-I is bound monomerically, as the cartoon shows. (**B**) Bar chart comparing *K*_D_ of RIG-I for various dsRNAs, measured using fluorescence polarization assays. 20 nM of RNA (either 5′ PPP ds39 RNA, 5′ NAD^+^ ds39 RNA, 5′ dephospho-CoA ds39 RNA, 5′ FAD ds39 RNA, or 5′ OH 5′ 2nt overhang RNA) and 0.5 mM of ATP were incubated with increasing concentrations of RIG-I and fluorescence polarization was measured. Each measured point is an average of 3 trials (*n* = 3). Each reaction was fit using a hyperbolic equation (Equations 2 and 3), and both *K*_D_ and error are derived from this fit. Because of high RIG-I concentrations relative to RNA, multiple RIG-I molecules are likely bound to RNA under this assay condition. Thus, the measured *K*_D_ by this assay is a composite one. (**C**) Electrophoretic mobility shift assay (EMSA) of RIG-I (0 nM in minus lanes, 75 nM in plus lanes) incubated with 25 nM of either 5′ PPP, 5′ alternative cap, or 5′ OVG 5′ OH RNA dsRNAs and 2 mM ATP. The sulfhydryl group of 5′ dephosphoCoA can form a disulfide linkage with another 5′ dephosphoCoA (note the higher band in the RNA alone lane). Monomer, dimer, and trimer RIG-I are denoted on the right of the gel, with cartoons. (**D**) Fluorescence polarization RNA competition assay confirming that RIG-I does not bind to single-stranded 5′PPP or 5′ NAD^+^ RNAs. A pre-mixed 50 nM RIG-I and 20 nM fluorescent 5′PPP dsRNA was added into serially diluted unlabeled ssRNA or 5′PPP dsRNA at 25°C in the presence of 500 uM ATP. The dashed line indicates the polarization value of free RNA.

We leveraged RIG-I’s ability to hydrolyze ATP when bound to RNA to determine the apparent *K*_D_ of RIG-I for each RNA. The ATPase assay informed whether metabolite-capped RNA could effectively bind and stimulate ATP hydrolysis by RIG-I, which is important for RIG-I signaling activity (30). Five dsRNAs were tested, each with a different 5′ end modification: 5′ triphosphate (5′ PPP), a PAMP RNA positive control; 5′ OH, 5′ 2-nt overhang (5′ OH, 5′ OVG), a negative control; and 5′ NAD^+^, 5′ FAD, and 5′ dephospho-CoA (5′ dpCoA), the metabolite-capped RNAs (Figure 3A, Supplemental Figure S3). The ATPase activity increased hyperbolically with the increase in RNA concentration, and the data fit a quadratic binding equation (Equation 1) to provide the apparent RNA K_D_ values. The 5' PPP and 5' NAD RNAs have an almost identical binding affinity with *K*_D, app_ ∼ 2 nM. The 5' dpCoA and FAD capped RNAs bind 2–3-fold more weakly than 5′ PPP but still bind with sub-nanomolar affinity (*K*_D, app_ ∼ 6–7 nM). The 5′ OH 5′ OVG binds with the weakest affinity (*K*_D, app_ ∼ 52 nM), 8-fold worse than the weakest binding metabolite-capped dsRNA, FAD. Additionally, each metabolite-capped dsRNA showed similar rates of ATP hydrolysis and comparable *V*_max_ values as 5′ PPP (Supplemental Figure S3), indicating that these are productively bound RIG-I/RNA complexes. These results indicate that metabolite-capped RNAs are recognized by RIG-I, like the Cap0 dsRNA (9), and with affinities similar to the well-established RIG-I PAMP 5′ PPP dsRNA. Additionally, metabolite-capped RNAs stimulate RIG-I’s ATPase to the same extent as the 5′PPP dsRNA.

Because the ATPase-based assay is an indirect method to measure RNA binding affinity, we also used fluorescence polarization assays that directly estimate the RNA *K*_D_ values (Figure 3B, Supplemental Figure S4). The same RNA panel was tested, and like the ATP hydrolysis assay results, RIG-I bound all metabolite-capped RNAs comparably to 5′ PPP dsRNA and between 3.5- and 6-fold worse than 5′ OH 5′ OVG dsRNA. Thus, two RNA binding methods confirm that RIG-I binds metabolite-capped dsRNAs indistinguishably from 5′ PPP dsRNAs.

Noticeably, all tested RNAs appeared to bind more weakly in polarization assays than in the ATP hydrolysis assays (compare the *K*_D_ and *K*_D, app_ of 5′ PPP of ∼50 and 1.5 nM, respectively). In the ATPase assay, we titrate a small amount of RIG-I with increasing RNA, conditions that support mostly RIG-I monomers on RNA. On the other hand, we titrate a small amount of RNA in the polarization assay with increasing RIG-I concentrations, conditions that support RIG-I oligomerization (Figure 3C). Thus, the RNA *K*_D_ values from the polarization assay are composite values of multiple RIG-I binding events rather than a single binding event in the ATPase assay. This explains the difference in *K*_D_s’ between the two assays.

With a footprint of approximately 10 bp per monomer (3), the 39-bp dsRNA can accommodate at least three RIG-I molecules, as shown by the EMSA results (Figure 3C). With a threefold excess RIG-I over RNA, all RNAs supported RIG-I dimers, and trimers were observed on the 5′ PPP and metabolite-capped dsRNAs. A higher-order disulfide-linked RNA dimer was observed with 5′ dpCoA dsRNA, deduced from the minus RIG-I lane and detected by MS. The long 5′OH 5′ OVG dsRNA also oligomerized RIG-I, but the amount of trimer was modest compared to the other RNAs tested.

We used an RNA competition assay to determine if RIG-I binds to 5′PPP or 5′ NAD^+^ ssRNAs. A complex of RIG-I and fluorescein-labeled 5′ PPP ds39 was mixed with increasing concentrations of unlabeled 5′PPP ds39 or 5′ PPP/5′ NAD^+^ ds39 ssRNA in the presence of ATP. While the ds39 competed effectively with the labeled RNA, the ssRNAs did not, demonstrating that RIG-I does not bind metabolite-capped ssRNAs noncanonically (Figure 3D).

### RIG-I can effectively utilize metabolite-capped dsRNAs as signaling ligands in HEK293T cells

**Figure 4.**
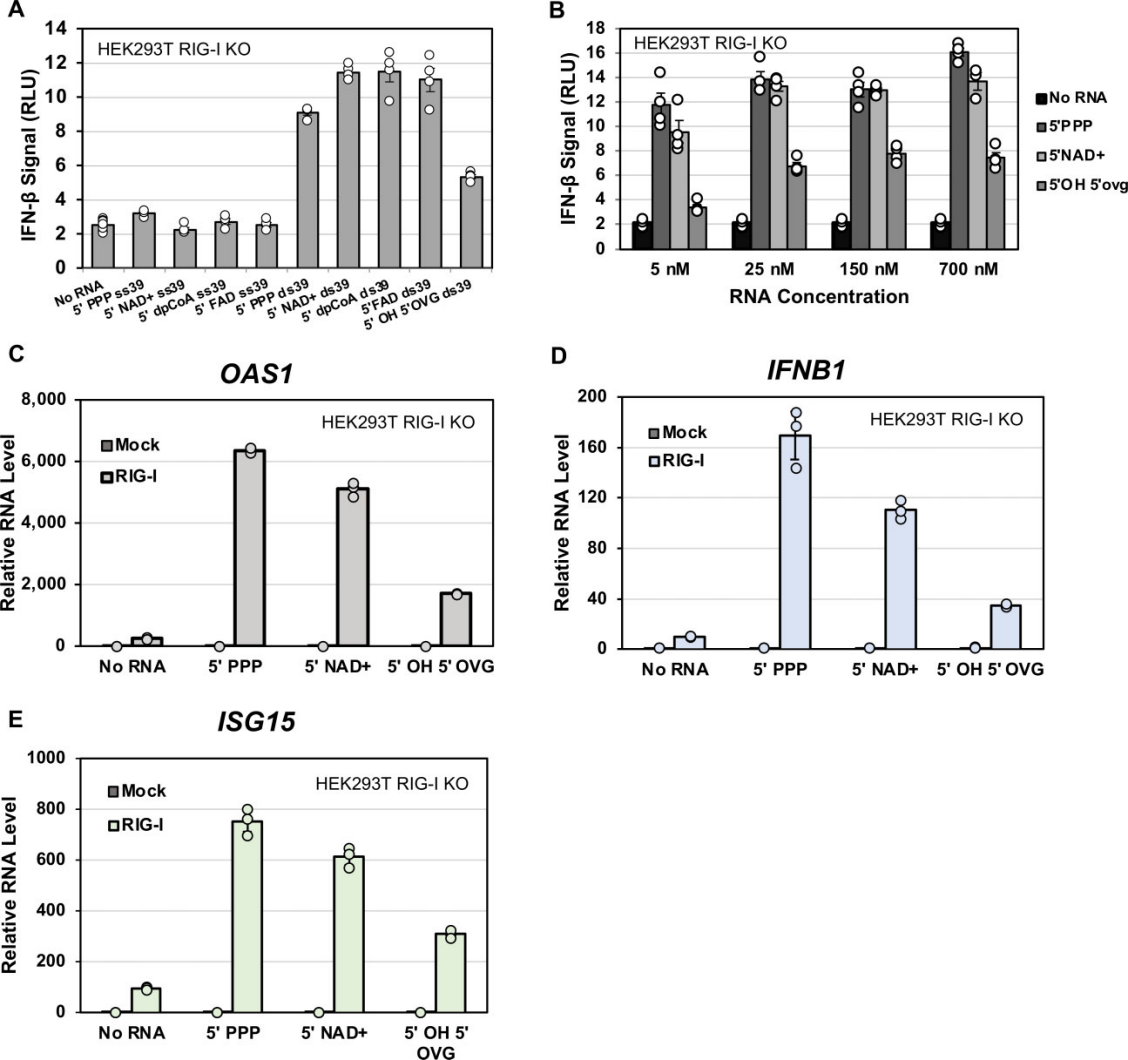
RIG-I utilizes metabolite-capped dsRNAs as potent signaling ligands. (**A**) HEK293T cells were transfected with RIG-I and a luciferase reporter assay system. Cells were transfected with either no RNA (2 biological repeats, 6 mechanical repeats each, *n* = 12) or 700 nM of the listed RNAs (2 biological repeats, 2 mechanical repeats each, *n* = 4). Dots indicate individual trials; bars indicate the average measurement, and error bars indicate SEM. (**B**) Dose-dependent titration of 5′ PPP, 5′ NAD^+^, and 5′ OH 5′ OVG RNAs to examine whether 5′ PPP and 5′ NAD^+^ signaled similarly at low RNA concentrations. The No RNA background (2 biological repeats, 3 mechanical repeats each, *n* = 6) average is included with each RNA concentration for reference. Each RNA titration average consisted of 2 biological repeats, 2 mechanical repeats each (*n* = 4). Dots represent individual trials minus average background, bars represent average measurement, and error bars represent average SEM. (**C**–**E**) HEK293T RIG-I KO cells with RIG-I or mock empty vector were transfected with the indicated ds39 RNA (50 nM) or no RNA. The extracted RNA was reverse transcribed into cDNA and analyzed by qPCR for expression of OAS1, IFNB, ISG15 and GAPDH mRNAs. The bar chart shows the ΔΔCt values normalized to the value of GAPDH. Each trial was done with three technical replicates (*n* = 3). Dots indicate individual trials; bars indicate the average measurement, and error bars indicate SEM.

Cellular reporter assays were used to assess whether the metabolite-capped RNAs could induce the expression of a luciferase reporter gene under the control of IFNβ promoter and thus function as a RIG-I signaling ligand. A mammalian expression vector with the RIG-I gene under a constitutively active promoter was transfected into HEK293T cells lacking endogenous RIG-I (31). RIG-I expression was verified by western blotting (Supplemental Figure S6A). RIG-I expressing cells were transfected with 5′ PPP dsRNA, 5′ OH 5′ OVG dsRNA, and the above-described panel of metabolite-capped dsRNAs. The metabolite-capped ssRNAs did not produce an interferon response above the background (Figure 4A). This is consistent with the weak affinity of RIG-I to ssRNA, reported earlier for 5′PPP ssRNA (32) and demonstrated here for metabolite-capped ssRNA by the competition assays (Figure 3D). This result also demonstrates that there was no copyback dsRNA in our sample generated from the *in vitro* transcription. In contrast to the ssRNAs, the dsRNAs with alternative caps signaled at levels comparable to 5′ PPP dsRNA. The 5′ OH 5′ OVG dsRNA, our negative control, signaled approximately threefold less than the capped or the 5′ PPP dsRNA, consistent with its poor binding to RIG-I.

A dose-dependent study, where 5′ PPP, 5′ NAD^+^ and 5′ OH 5′ OVG dsRNAs were titrated from 5 to 700 nM, was conducted to assess the relative signaling potency of these RNAs. The signaling activity of all tested RNAs remained constant between 25 and 700 nM, with 5′ OH 5′ OVG consistently signaling approximately 2.5-fold lower than 5′ PPP or 5′ NAD^+^ (Figure 4B). At 5 nM RNA concentration, the 5′ OVG-dependent signaling decreased by 4-fold relative to higher RNA concentrations, consistent with its weaker affinity for RIG-I, while 5′ PPP and 5′ NAD^+^ dsRNA-dependent signaling did not significantly decrease. These results indicate that RIG-I recognizes NAD^+^ capped RNA with a similar sensitivity as the well-established PAMP, 5′ PPP RNA, which agrees with their similar RNA binding properties.

To determine if the metabolite-capped RNAs can activate the expression of endogenous interferon and antiviral response-related transcripts, we used RT-qPCR to measure the mRNA levels of IFNB1 (Interferon beta 1), OAS1 (2′-5′-oligoadenylate synthetase 1) and ISG15 (Interferon stimulated gene 15) after ectopic expression of RIG-I in HEK293T RIG-I KO cells (Figure 4C–E). In all tested cases, 5′ PPP and 5′ NAD^+^ ds39 RNAs stimulated comparable OAS1, IFNB1 and ISG15 transcription levels, 8–25-fold higher than the no RNA background. Additionally, 5′ OH 5′ OVG ds39 signaled approximately 2–3-fold worse than 5′ PPP or 5′ NAD+, consistent with the reporter assay results. These results demonstrate that RIG-I effectively recognizes metabolite-capped RNAs as immunogenic ligands in cellular contexts.

### Endogenous RIG-I in A549 cells generates an innate immune response against metabolite-capped RNAs

**Figure 5.**
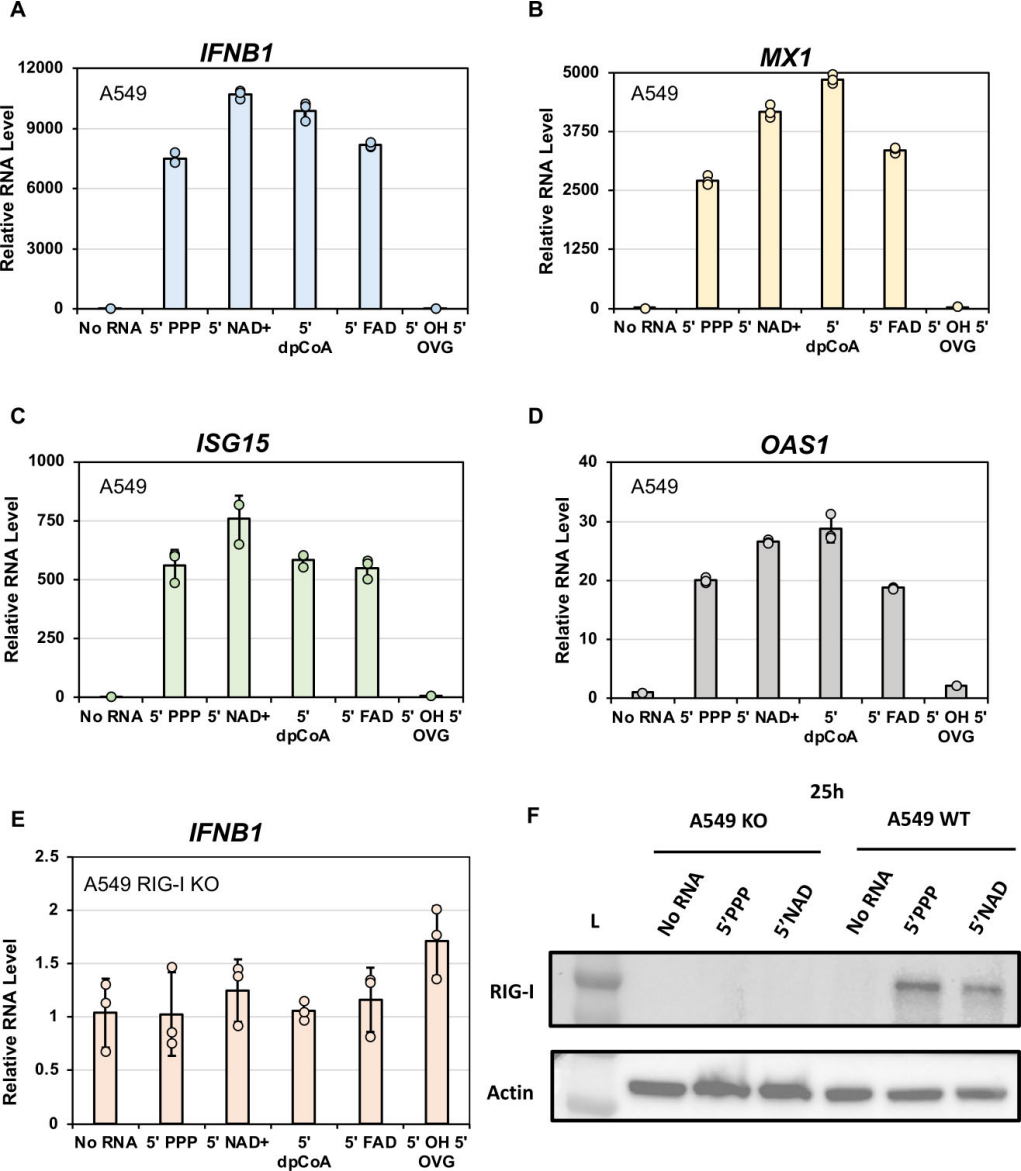
Endogenous RIG-I in A549 cells utilizes metabolite-capped dsRNAs as potent signaling ligands. (**A–D**) A549 cells and (E) A549 RIG-I KO cells were transfected with the indicated ds39 RNA (100 nM) or no RNA. The extracted RNA was reverse transcribed into cDNA and analyzed by qPCR for OAS1, IFNB, MX1, ISG15 and GAPDH mRNA expressions. The bar chart shows the ΔΔCt values normalized to the value of GAPDH. Each trial was done with three technical replicates (*n* = 3). Dots indicate individual trials; bars indicate the average measurement, and error bars indicate SEM. (**F**) Western blot for A549 RIG-I KO and WT cells transfected with 100 nM 5'PPP 5'NAD^+^ds39 RNA and no RNA probed with anti-RIG-I and anti-actin antibody.

The reporter signaling assay, shown above, relied on overexpressed, ectopic RIG-I. To determine if endogenous levels of RIG-I would recognize the metabolite-capped RNAs, we used A549 cells. In agreement with our reporter assay in HEK293T, the 5′ PPP ds39 and all the metabolite-capped RNAs generated a robust antiviral signaling state in A549 cells, showing expression of IFNB1, OAS1, ISG15, and MX1 (MX dynamin-like GTPase 1) genes (Figure 5A–D). In addition, Western blotting confirmed that RIG-I expression significantly increased in the presence of 5′ PPP and metabolite-capped RNAs (Supplemental Figure S7). Interestingly, with a lower level of RIG-I in A549 cells, the 5′ OVG 5′ OH dsRNA signaled very poorly, comparably to no RNA transfected controls, and did not stimulate endogenous RIG-I expression. In contrast, when RIG-I was ectopically expressed in HEK293T experiments, this RNA generated an immune signal greater than the no RNA control but still less than the rest of the panel.

To confirm that the immune stimulation observed in A549 cells was RIG-I-dependent, we transfected A549 RIG-I KO cells with our RNA panel, monitoring the antiviral immune response using qPCR targeting IFNB1 (Figure 5E). Agreeing with our hypothesis, none of the RNAs could stimulate antiviral signaling in RIG-I KO cells, demonstrating that the recognition of these RNAs is RIG-I dependent. Our study reveals that metabolite-capped dsRNAs are RIG-I ligands that activate the interferon and antiviral immune signaling pathways, representing a new class of RIG-I stimulatory RNAs.

## DISCUSSION

The cellular machinery of the higher eukaryotes adds an m^7^G RNA cap to a mature mRNA with Cap1 modification to evade RIG-I recognition and prevent an immune response (9, 10). Alternative, metabolite-based 5′ RNA caps, such as NAD^+^, FAD and dephosphoCoA, are found in various organisms, including human cells where RIG-I is present (18–20). Whether RIG-I recognizes these newly identified caps as immunogenic or these caps confer protection against RIG-I recognition has important implications for the functions of metabolite-derived RNA caps, as well as illustrating the mechanisms behind RIG-I’s interaction with such RNAs. This study shows that RIG-I recognizes the metabolite-capped RNAs as immunogenic, similar to 5′ triphosphorylated and Cap0 RNAs (9, 10). RIG-I binds to, hydrolyzes ATP on, and can oligomerize on these metabolite-capped RNAs—all essential biochemical functions for activating the RIG-I signaling pathway (30). Cellular assays show that endogenous and ectopically expressed RIG-I can utilize these metabolite-capped RNAs as signaling ligands to stimulate the antiviral immune signaling pathway. Animal studies are required to confirm the immunogenicity of metabolite-capped RNAs *in vivo*.

This study also describes a strategy to effectively transcribe metabolite-capped RNAs using the T7 RNAP system without purifying out contaminating 5′ PPP RNA and copyback transcripts. First, the initiating nucleotide, A, is only present at the +1 position, which removes the need to add ATP. Secondly, the terminal two nucleotides of the template strand are methylated to promote T7 RNAP dissociation and reduce nonspecific 3′ end extension and copyback transcription, resulting in clean and precise RNA ends. Finally, NTPs complementary to the terminal dinucleotide are excluded from the transcription reaction to inhibit copyback transcription. While these sequence constraints could be prohibitive for transcribing long RNAs or RNAs whose sequences are meant to mimic those found *in vivo*, this strategy does allow clean and convenient transcription of smaller metabolite-capped RNAs. The ADP-based metabolites system demonstrated here could be adapted to other metabolites with different NDP substructures, like UDP-GlcNAc, or to artificially synthesized NDP-based moieties, with a concomitant change in RNA sequence while preserving the key design elements.

The study of RIG-I cap recognition so far has been limited to the role of the canonical m^7^G cap. This study demonstrates that RIG-I has a broad tolerance for bulky groups with disparate geometries at the 5′ end of the RNA with at least a diphosphate backbone between the cap and the first RNA nucleotide. A previously published crystal structure of RIG-I bound to Cap0 dsRNA (m^7^G-capped RNA) (9), can be used to understand a potential mechanism of metabolite-capped RNA recognition by RIG-I. In this structure, CTD of RIG-I interacted exclusively with the triphosphate linkage without making noticeable interactions with the Cap0 moiety. By overlaying multiple members of the asymmetric unit, it was inferred that the m^7^G cap is flexibly accommodated in the CTD binding pocket without specific contacts (Figure 1A). Likely, this is also occurring with the metabolite-capped dsRNAs. While metabolite-capped RNAs contain a diphosphate rather than triphosphate linkage between RNA and cap moiety, the predominant interactions between 5′ PPP and CTD are with the α- and β-phosphates (3, 9). The potential change in spatial orientation of the alternative cap due to the lack of a third phosphate linkage is not as crucial as the diphosphate linkage. Thus, it is likely that CTD binds to the diphosphate linkage without making important contacts with the metabolite cap moieties. Interestingly, the tested caps have a range of geometries: NAD^+^ is similar in size to m^7^G canonical cap, dephosphoCoA has a long hydrocarbon chain, and FAD has a bulky, three-membered ring structure. The finding that large functional groups can be added to the 5′ end of RNAs and still be recognized by RIG-I can be leveraged for RNA technologies that rely on RIG-I, either avoiding it (requiring additional modifications) or triggering it (5′ end modifications imparting new functionalities to the RNA) (33).

The role of the metabolite-capped RNAs in mammals is not well understood, but if such RNAs accumulate in the cytoplasm of healthy cells, RIG-I will recognize them as immunogenic. Sensitive RIG-I recognition of metabolite-capped RNAs that are endogenously made needs to be demonstrated. Still, if they are immunogenic, additional mechanisms would be required to prevent deleterious autoimmune responses without viral infection. Such mechanisms could include internal RNA modifications, including methylation to inhibit RIG-I signaling (30, 34) or methylating the 2′-*O*-ribose like in Cap1 RNA. However, whether existing methylation pathways work with metabolite-capped RNAs is unknown and needs further investigation. Other means to evade RIG-I could include preventing dsRNA formation, sequestering the 5′ RNA end through protein binding, burying the RNA group in a larger structure, such as the ribosome, or sequestering the RNA in organelles outside the cytoplasm, such as the mitochondria or nucleus. When leaking from damaged mitochondria or suffering mitochondrial DNA damage, human mitochondrial RNAs enter the cytoplasm and stimulate RIG-I and MDA5 (19, 35, 36). Mitochondrial RNAs are not capped by m^7^G but have been observed to incorporate alternative caps (19). The appearance of RIG-I-stimulatory metabolite-capped RNAs in the cytoplasm may represent an important sensor for mitochondrial health. Whether alternative capping imparts additional RIG-I recognition functions or imposes different RNA regulatory mechanisms is unknown.

An understudied context of alternative capping is the viral capacity for using metabolites to cap their RNAs. Only one description of virally derived, metabolite-capped RNAs is found in Dengue virus-infected mosquito cells (18). Dengue virus synthesizes a canonical m^7^G capped mRNA for protein translation and RIG-I evasion (37). Additionally, while the Dengue virus has an initiating A in its genome, the most abundant metabolite-capped RNA found was UDP-GlcNAc, suggesting possible preferential misincorporation during initiation facilitated by a U–U wobble base pair. It would be pertinent to test for virally derived, metabolite-capped RNAs in other organism-derived cell lines (such as humans rather than mosquitoes) and in other viruses, such as other flaviviruses and unrelated RNA viruses. Additionally, it may be essential to examine whether metabolite-capped RNAs are associated with defective interfering genomes (DIs), which are known to be highly immunogenic (38). Recognition of viral metabolite-capped RNAs may represent a metabolite-mediated host defense mechanism, whereby viral RNA polymerase misincorporates NTP-similar metabolites, leading to mRNA-capping-incompetent RNA that cannot be translated in the cytoplasm but robustly triggers RIG-I.

Altogether, the discovery that RIG-I recognizes a broader range of capped RNAs than previously known m^7^G cap presents additional constraints on how metabolite-capped RNAs can exist in the cytoplasm of a healthy cell and new avenues to explore their applications for RNA-based technologies.

## Supplementary Material

gkad518_Supplemental_File

## Data Availability

ATP hydrolysis-based binding studies, electrophoretic mobility shift assay data, cell signaling reporter data, RT-qPCR data, and Western blots for main figures have been deposited to the BioStudies database (https://www.ebi.ac.uk/biostudies/) and assigned the identifier S-BSST911.
