# Correction to ‘RIG-I recognizes metabolite-capped RNAs as signaling ligands’

**DOI:** 10.1093/nar/gkaf933

**Published:** 2025-09-12

This is a correction to: Brandon D Schweibenz, Mihai Solotchi, Pranita Hanpude, Swapnil C Devarkar, Smita S Patel, RIG-I recognizes metabolite-capped RNAs as signaling ligands, *Nucleic Acids Research*, Volume 51, Issue 15, 25 August 2023, Pages 8102–8114, https://doi.org/10.1093/nar/gkad518

The published version of this manuscript omitted to note that Brandon D Schweibenz and Mihai Solotchi contributed equally and should be considered co–first authors.

These details have been corrected only in this correction notice to preserve the published version of record.

