# Supplementary material for: RIG-I recognizes metabolite-capped RNAs as signaling ligands
Source: Nucleic Acids Res. 2023 Jun 16;51(15):8102–14. doi: 10.1093/nar/gkad518 (PMC10450190; doi:10.1093/nar/gkad518)
Supplement: gkad518_Supplemental_File [file gkad518_supplemental_file.pdf]

Oligo	Sequence
T7 Transcription ss39 nontemplate DNA	5'- CAG TAA TACGAC TCA CTA TTA GTT GGT GGT TGT TGT GTG TTT GTG GTT GGT TTG TTT GG -3'
T7 Transcription ss39 template DNA	5'- GTC ATT ATG CTG AGT GAT AAT CAA CCA CCA ACA CAC AAA CAC CAA CCA AAC AAA CC -3'
Transcribed ss39 RNA	5'- XGU UGG UGG UUG UUG UGU GUU UGU GGU UGG UUU GUU UGG -3'
Complementary ss39 RNA	5'- dT(Fam-T)dT CCA AAC AAA CCA ACC ACA AAC ACA CAA CAA CCA CCA ACU -3'
5' OH 5' OVG ss41 RNA	5'- AG AGU UGG UGG UUG UUG UGU GUU UGU GGU UGG UUU GUU UGG -3'
5' PPP ss14 chimera RNA	5'- CGU GAG ACA UA dGdCdG -3'
Complementary ss14 chimera RNA	5'- dCdGdC UA UGU CUC ACG -Fl-3'
5' PPP ss27 RNA	5'- AUA CGU CCU GAU AGU UAG UAU CCA UCG -3'
Complementary ss27 RNA	5'- Biotin – CGA UGG AUA CUA ACU AUC AGG ACG UAU – DY547 -3'
GAPDH Forward Primer	5'- TCTCTGCCCCCTCTGCTG -3'
GAPDH Reverse Primer	5'- AGTCCTTCCACGATACCAAA -3'
IFNB Forward Primer	5'- GGGACTGGACAATTGCTTCAA -3'
IFNB Reverse Primer	5'- GCAGTACATTAGCCATCAGTCACTTAA -3'
ISG15 Forward Primer	5'- GAGAGGCAGCGAACTCATCT -3'
ISG15 Reverse Primer	5'- CTTCAGCTCTGACACCGACA -3'
OAS1 Forward Primer	5'- GAAGGAAAGGTGCTTCCGAGGTAG -3'
OAS1 Reverse Primer	5'- AAGACAACCAGGTCAGCGTCAGAT -3'
MX1 Forward Primer	5'- CTGGGATTTTGGGGCTTT -3'
MX1 Reverse Primer	5'- GGGATGTGGCTGGAGATG -3'

Supplemental Table 1. Oligonucleotide sequences used in this study. Transcribed ss39 RNA was in vitro transcribed, and the 5' 'X' indicates that this position could either be a 5' PPP or a metabolite cap. DNA nucleotides are indicated with a preceding "d" in RNA oligonucleotides. "Fam-T" indicates a T labeled with fluorescein. 3' "Fl" indicates fluorescein.

A

T7 promoter region | Transcript region

```
NT 5′ - CAG TAA TAC GAC TCA CTA TTA GTT GGT GGT TGT TGT GTG TTT GTG GTT GGT TTG TTT GG - 3′
T  3′ - GTC ATT ATG CTG AGT GAT AAT CAA CCA CCA ACA ACA CAC AAA CAC CAA CCA AAC AAA CC - 5′
```

B

```
5′ - XGU UGG UGG UUG UUG UGU GUU UGU GGU UGG UUU GUU UGG - 3′
```

C

D

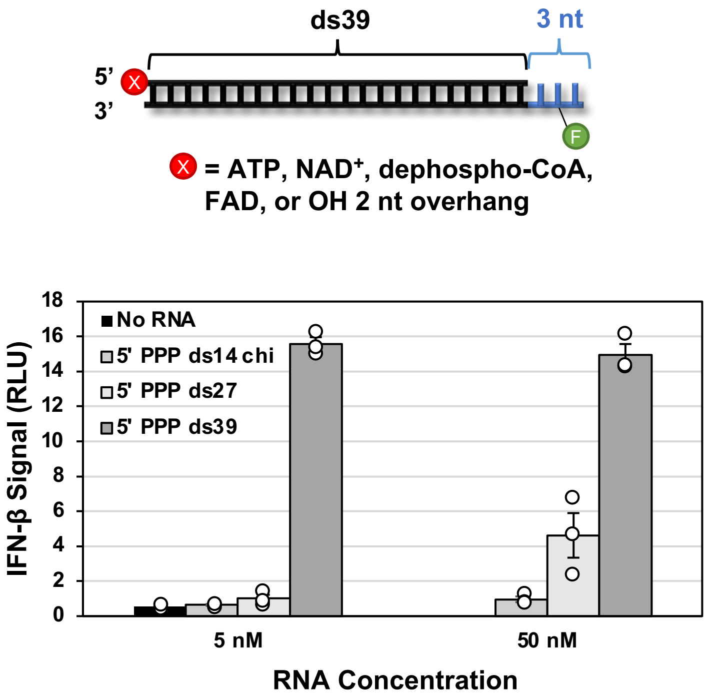

Supplemental Figure 1, Related to Figure 2.
(**A**) The sequence of the T7 DNA promoter. NT stands for the Nontemplate strand, and T stands for the Template strand for transcription. The NT strand is the resulting RNA sequence, with U instead of T. The bold A is the transcription start site.
(**B**) Sequence of the RNA transcribed from the template in (A). Note that the first position has been labeled with an X in red to indicate that the first position can be ATP or the metabolite, depending on the nucleotide added to the transcription reaction.
(**C**) Design of transcribed, metabolite-capped ds39 RNAs used in this study. Black lines indicate RNA, while blue lines indicate a 3-nt blue DNA overhang distal to the cap of interest. The 2nd nucleotide of the DNA overhang is labeled with fluorescein.
(**D**) RIG-I signaling ability compared to RNA length in HEK293T cells. HEK293T cells were transfected with RIG-I and a luciferase reporter assay system. Cells were transfected with either no RNA (3 technical repeats), and then 5 nM or 50 nM of the listed RNAs (3 technical repeats each). Dots indicate individual trials, bars indicate the average measurement, and error bars indicate SEM. RNAs tested all contained blunt-ended, 5′ PPP dsRNA features. ds14 chi has 4 bp of chimeric DNA at the 5′ PPP distal end, blocking RIG-I binding. ds27 has 27 RNA base pairs, with a 5′ 3 nt DNA overhang distal to the 5′ PPP end. ds39 is described in (**C**). RNA sequences used can be found in **Supplemental Table 1**.

A **5’ PPP**

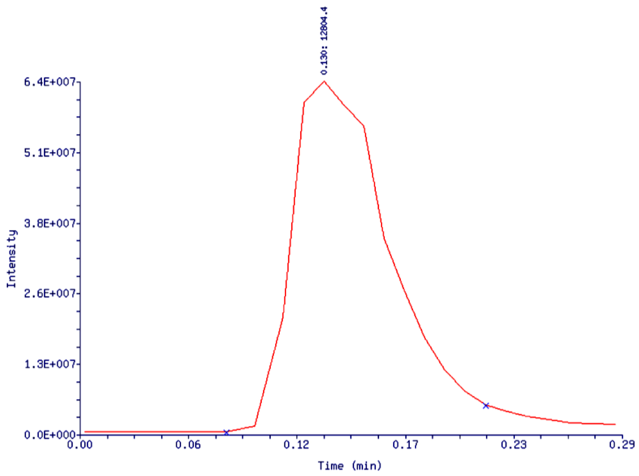

Peak Mass (Da)	MS Peak Area	MS Peak Percent
12804.4	2.16×10^{8}	100

B **5’ NAD⁺**

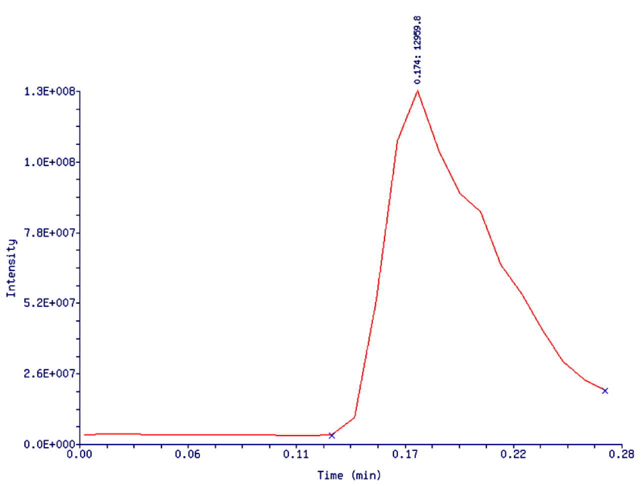

Peak Mass (Da)	MS Peak Area	MS Peak Percent
12959.8	4.30×10^{8}	100

C **5’ dephospho-CoA**

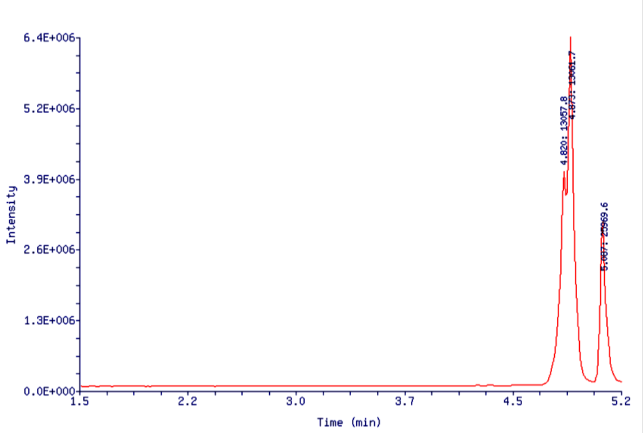

Peak Mass (Da)	MS Peak Area	MS Peak Percent
13057.8	5.38×10^{5}	40.34
13061.7	6.32×10^{5}	47.42
25969.6	1.63×10^{5}	12.24

D **5’ FAD**

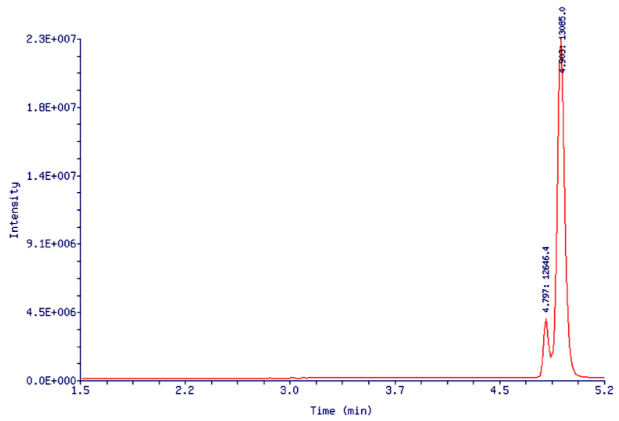

Peak Mass (Da)	MS Peak Area	MS Peak Percent
12646.4	2.85×10^{5}	10.80
13085.0	3.26×10^{6}	89.20

Supplemental Figure 2, Related to Figure 2.

MS curves for 5’ PPP (A), NAD^{+} (B), FAD (C), and dephospho-CoA (D). Below each curve is a table describing the peaks. Note that each RNA was assayed for purity asynchronously and under different methods; thus, each RNA’s x-axis (retention time) is not directly comparable.

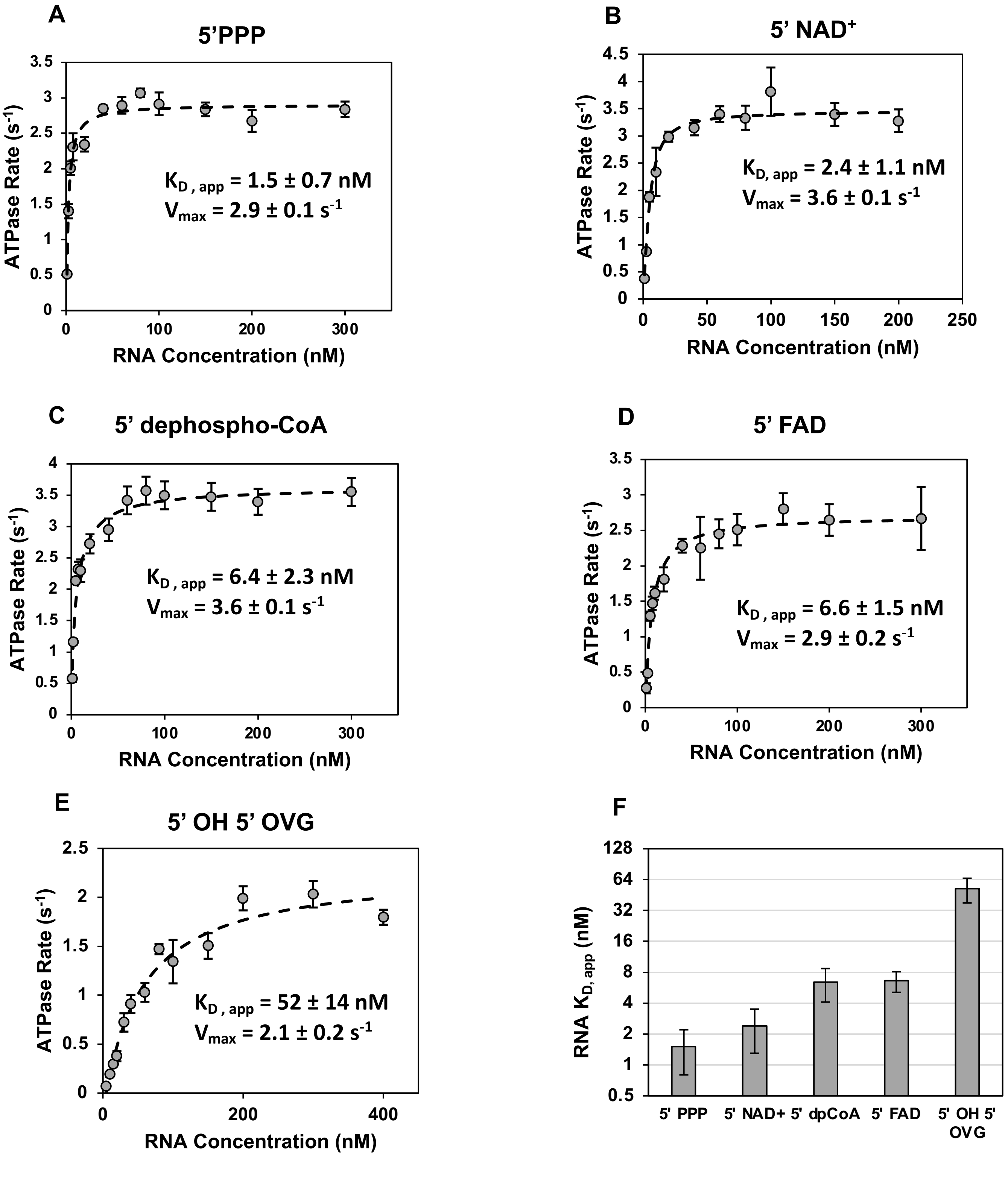

Supplemental Figure 3, related to Figure 3. RIG-I can bind to and hydrolyze ATP using metabolite-capped RNAs effectively.

(**A**) – (**E**) RIG-I (15 nM) was incubated with 2 mM ATP spiked with [γ-^{32}P]-ATP, 1x ATPase Buffer, and increasing concentrations of either (A) 5' PPP ds39 RNA, (B) 5' NAD^{+} ds39 RNA, (C) 5' dephospho-CoA ds39 RNA, (D) 5' FAD ds39 RNA, or (E) 5' OH 5' 2nt overhang RNA. Each point represents an individual reaction run with time points of either 0", 20", 40", or 60" and fit with a linear equation. Error bars are from each linear fit of three technical replicates per point (n = 3). Each overall reaction was fit using a quadratic equation (dashed lines, Equation 1), and both $K_{D, app,}$ and V_{max} were derived from this fit. The fit determined errors for these two values.

(**F**) Bar graph compilation of $K_{D, app}$ data shown in (A) – (E). Note the $\log_2$ y-axis scale.

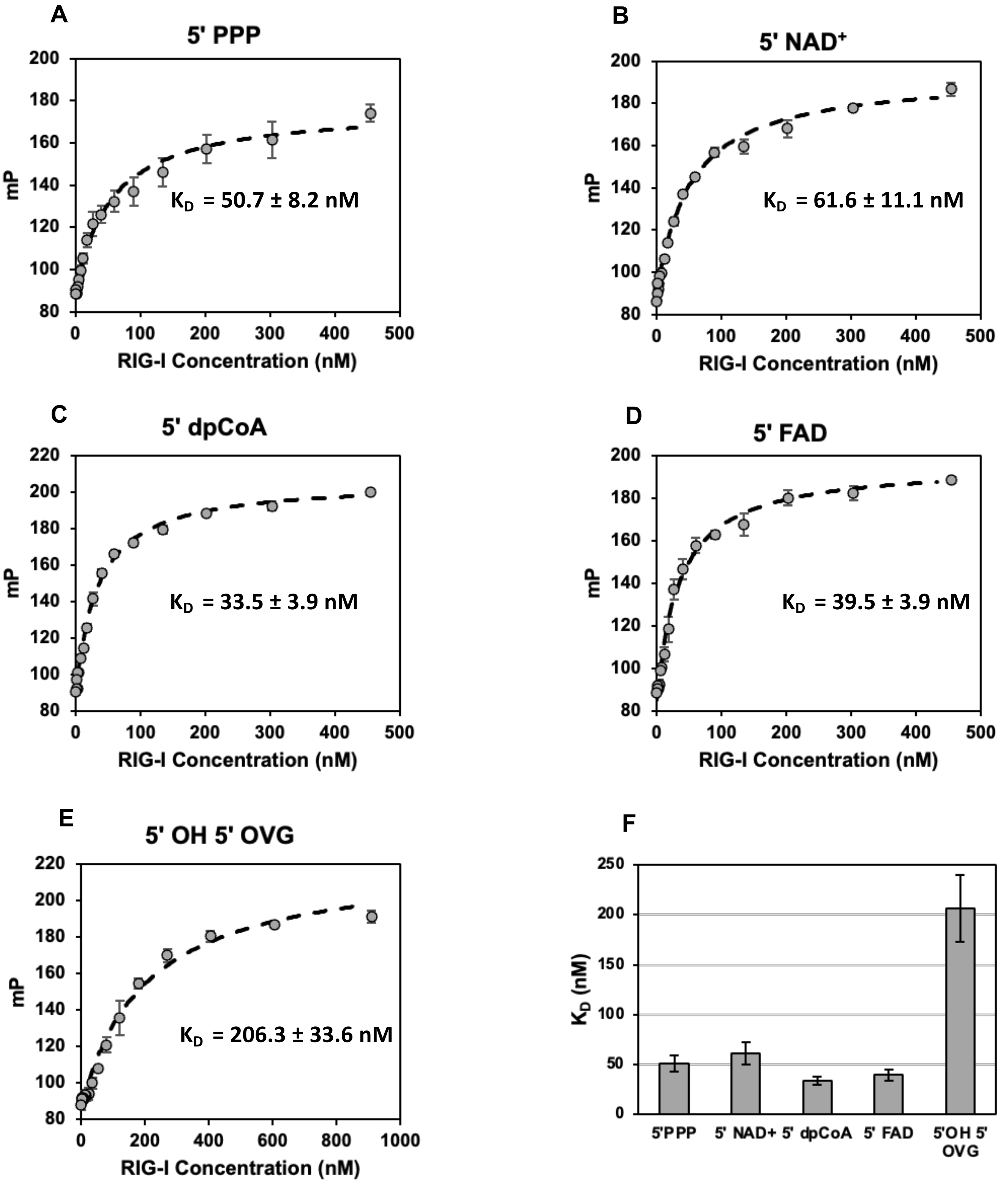

Supplemental Figure 4, related to Figure 3. RIG-I directly binds metabolite-capped RNAs comparably to 5' PPP.
(**A**) – (**E**) Fluorescein-labelled RNA (20 nM), either (A) 5' PPP ds39 RNA, (B) 5' NAD^+ ds39 RNA, (C) 5' dephospho-CoA ds39 RNA, (D) 5' FAD ds39 RNA, or (E) 5' OH 5' 2nt overhang was incubated with 0.5 mM ATP, 1x ATPase Buffer and increasing concentrations RIG-I, and fluorescence polarization was measured (mP). Each point represents an average of three trials; error bars are the standard error of these three trials (n=3). Each overall reaction was fit using a hyperbolic equation (dashed lines, Equation 2 and 3) to estimate the K_D values.
(**F**) Bar graph compilation of K_D data shown in (A) – (E). Error bars are from each fit of three technical replicates per point (n = 3).

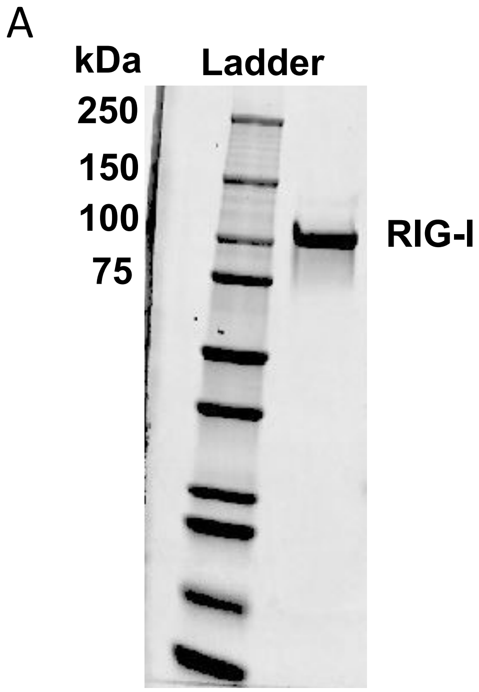

Supplementary Figure 5, related to Figure 3.

(A) Purified full-length RIG-I was used in this study. The predicted mass of full-length RIG-I is 106.6 kDa.

A

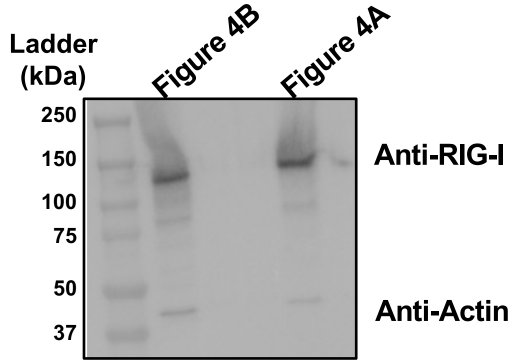

B

Reverse transcription reaction- HEK293T RIG-I KO							
Label#	Plasmid	RNA	RNA Stock	Vol. of RNA	Vol. of H_2O	Total RNA for RT in 10µl	2 x RT Master Mix
			(ng/µl)	(µl)	(µl)	(ng)	(µl)
**R01**	mock EV	No RNA	1112.6	1.35	8.65	1500	10
**R02**	mock EV	5’PPP ds39	1253.2	1.20	8.80	1500	10
**R03**	mock EV	NAD^+-ds39	1348.6	1.11	8.89	1500	10
**R04**	mock EV	5’OVG 5’OH ds39	1089.4	1.38	8.62	1500	10
**R05**	WT RIG-I Myc	No RNA	1539.6	0.97	9.03	1500	10
**R06**	WT RIG-I Myc	5’PPP ds39	1362	1.10	8.90	1500	10
**R07**	WT RIG-I Myc	NAD^+-ds39	760.7	1.97	8.03	1500	10
**R08**	WT RIG-I Myc	5’OVG 5’OH ds39	708.3	2.12	7.88	1500	10

2 x RT Master Mix Components	Volume (µl)
10 x RT Buffer	2
25 x dNTP Mix (100 mM)	0.8
10 x RT Random primers	2
MultiScribe Reverse Transcriptase	1
Nuclease free H_20	4.2
**Total per reaction**	10

cDNA Synthesis			
Step1	Step2	Step3	Step4
25 ^{0}C	37 ^{0}C	85 ^{0}C	4 ^{0}C
10 min	120 min	5 min	hold

Supplemental Figure 6, Related to Figure 4.
(**A**) Western blot confirming expression of myc-tagged RIG-I in cell signaling reporter assays (Figures 4A and 4B).
(**B**) Tables of the reverse transcription reaction mixtures and cDNA synthesis PCR condition for total RNA extracted from HEK293T RIG-I KO cells transfected with EV and Myc-tagged RIG-I.

A

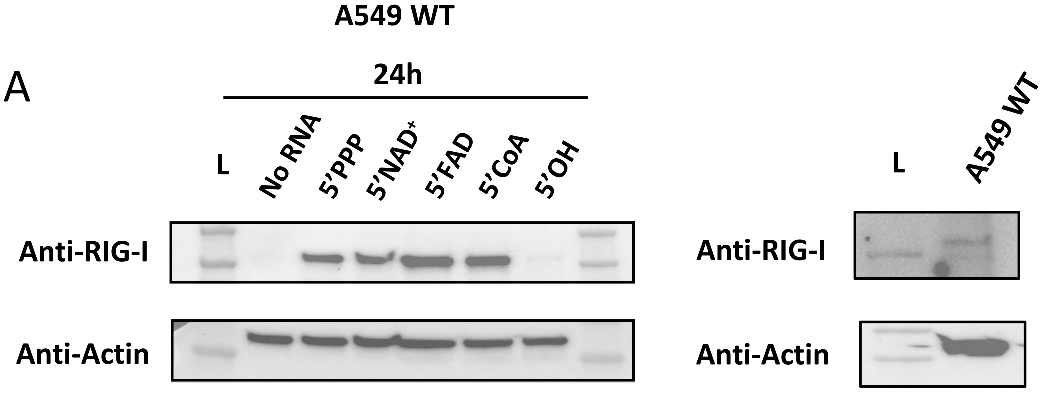

B

Reverse transcription reaction -A549 cells							
Label#	Cells	RNA	Stock RNA Conc.	Vol. of Stock RNA	Vol. of H2O	Total RNA f or RT in 10ul	2X RT Master Mix
		Condition	(ng/ul)	(ul)	(ul)	(ng)	(ul)
R01	A549 WT	No RNA	819.3	1.83	8.17	1500	10
R02	A549 WT	5'PPP ds39	708.5	2.12	7.88	1500	10
R03	A549 WT	5' NAD^+ ds39	821.5	1.83	8.17	1500	10
R04	A549 WT	5' dpCoA ds 39	711	2.11	7.89	1500	10
R05	A549 WT	5' FAD ds39	834.3	1.80	8.20	1500	10
R06	A549 WT	5' OH 5' OVGds39	865.9	1.73	8.27	1500	10
R07	A549 RIGI KO	No RNA	976.7	1.54	8.46	1500	10
R08	A549 RIGI KO	5'PPP ds39	914.7	1.64	8.36	1500	10
R09	A549 RIGI KO	5' NAD^+ ds39	959.4	1.56	8.44	1500	10
R10	A549 RIGI KO	5' dpCoA ds 39	984.3	1.52	8.48	1500	10
R11	A549 RIGI KO	5' FAD ds39	955	1.57	8.43	1500	10
R12	A549 RIGI KO	5' OH 5'OVGds39	892.7	1.68	8.32	1500	10

Supplemental Figure 7, Related to Figure 5.
(**A**) Western blot confirming expression of endogenous RIG-I in A549 cell after 24 h RNA transfection (Figure 5). Fresh antibody dilution of 1:500 was used, and the blot was overexposed to visualize the trace amount of endogenous RIG-I. (**B**) Table shows total RNA extracted and reverse transcription reaction mixture conditions. cDNA synthesis reaction mixture and PCR conditions are the same as in Supplemental Figure 6B.
